# Overall and domain-specific neuropsychiatric symptoms in relation to caregiver burden among family caregivers of individuals with cognitive impairment

**DOI:** 10.3389/fpsyt.2026.1937017

**Published:** 2026-09-30

**Authors:** Xuan Yang, Jinli Wang, Na Wei, Kaiting Fan, Hong Chen, Yuchen Qiao

**Affiliations:** 1 Department of Neurology, Xuanwu Hospital, Capital Medical University, Beijing, China; 2 School of Nursing, Capital Medical University, Beijing, China

**Keywords:** caregiver burden, cognitive impairment, dementia, family caregivers, neuropsychiatric symptoms

## Abstract

**Background:**

Neuropsychiatric symptoms (NPS) are common across the continuum of cognitive impairment and represent a major source of distress for family caregivers. Because NPS often co-occur in real-world care settings, it remains clinically important to determine whether caregiver burden is more closely related to cumulative NPS burden or to specific symptom domains.

**Objective:**

This study aimed to describe the prevalence and distribution of NPS and to examine the associations of Neuropsychiatric Inventory (NPI) total score, symptom count, caregiver-distress total score, and individual NPI domain scores with caregiver burden among Chinese patient-caregiver dyads with cognitive impairment.

**Methods:**

This single-center cross-sectional study included 418 patient–caregiver dyads recruited from the Neurodegenerative and Cognitive Disorders inpatient unit of Xuanwu Hospital, Capital Medical University. NPS were assessed using the NPI and caregiver burden using the ZBI. Associations between global and domain-level NPI measures and ZBI total score were examined using Spearman correlations and multivariable linear regression adjusted for patient clinical characteristics, cognitive and functional status, and caregiver-related factors. Domain-level P values were corrected using the Benjamini–Hochberg false discovery rate. Additional analyses separately modeled the 12 domain-specific frequency and severity ratings and examined within-domain frequency-by-severity interactions. Multiple imputation and sensitivity analyses were used to assess robustness.

**Results:**

At least one NPS was present in 251 patients (60.0%; 95% CI, 55.3%–64.6%). The most common symptoms were apathy/indifference (30.4%), depression/dysphoria (25.1%), anxiety (23.0%), nighttime behaviors (21.1%), and irritability/lability (18.7%). Among the 403 dyads with complete observed ZBI data, ZBI total score was correlated with NPI total score (Spearman ρ = 0.308), symptom count (ρ = 0.313), and caregiver-distress total score (ρ = 0.336; all P < 0.001). In the multiple-imputation analysis including all 418 dyads, higher NPI total score remained associated with higher ZBI total score after multivariable adjustment (B = 0.357; 95% CI, 0.236–0.478; standardized β = 0.339; P < 0.001), with similar associations for symptom count and caregiver-distress total score. No individual NPI domain remained significant after simultaneous adjustment and FDR correction. In co-occurrence analyses, the number of other positive NPI domains was consistently associated with higher ZBI scores. In the domain-level frequency, severity, and within-domain interaction analyses, no association remained significant after FDR correction. Sensitivity analyses were consistent with the primary findings.

**Conclusion:**

NPS were common among individuals with cognitive impairment and were associated with greater caregiver burden. Cumulative NPS measures showed more consistent associations with caregiver burden than individual symptom domains. These findings support comprehensive assessment of overall neuropsychiatric symptom burden when evaluating family caregiver burden.

## 1 Introduction

Cognitive impairment has become a major public health challenge for health systems worldwide. The number of people living with dementia is expected to increase substantially in the coming decades, mainly because of population aging and growth (1). In China, a nationwide cross-sectional study estimated that 15.07 million people aged 60 years or older were living with dementia and 38.77 million with mild cognitive impairment (MCI), highlighting the considerable clinical and social burden of cognitive disorders in an aging population (2). Individuals with cognitive impairment, particularly those with moderate to severe dementia, often require long-term medical, psychological, and social support, which places substantial demands not only on healthcare systems but also on family caregivers who provide daily care.

Clinically, cognitive impairment is commonly conceptualized along a continuum from MCI to dementia. MCI is generally characterized by objective cognitive impairment greater than expected for age and education, with relative preservation of independence in basic activities of daily living (3). Dementia, by contrast, involves progressive cognitive deterioration sufficient to interfere with daily functioning and is frequently accompanied by behavioral and psychological changes. Major etiological subtypes include Alzheimer’s disease, vascular dementia, dementia with Lewy bodies, frontotemporal dementia, and mixed dementia. Accordingly, the consequences of cognitive impairment extend to functional, behavioral, emotional, and caregiving domains.

Behavioral and psychological symptoms of dementia (BPSD), also referred to as neuropsychiatric symptoms (NPS), comprise a heterogeneous set of disturbances in mood, perception, thought, behavior, sleep, and appetite (4). Common symptoms include depression, anxiety, apathy, irritability, agitation or aggression, psychosis, disinhibition, aberrant motor behavior, sleep disturbance, and appetite changes. These symptoms are not limited to advanced dementia; they may emerge during the MCI stage and may precede or accompany cognitive impairment (5). Recent evidence suggests that specific symptoms, particularly apathy, may be associated with an increased risk of progression from MCI to dementia (6). Among people with dementia, NPS are highly prevalent and vary according to dementia subtype, disease severity, symptom definition, and care setting. This heterogeneity underscores the importance of characterizing both overall and domain-specific NPS across the spectrum of cognitive impairment.

Caregiver burden is a multidimensional construct encompassing the psychological, physical, social, and financial strain perceived by individuals who provide sustained care to a family member with cognitive impairment (7, 8). High caregiver burden may adversely affect caregivers’ mental and physical health, social functioning, quality of life, and the sustainability of home-based care (9, 10). NPS may be particularly burdensome because their recurrent or unpredictable nature can increase supervision and behavioral-management demands, disrupt daily routines and sleep, and contribute to emotional strain (10, 11).

Previous studies have shown that caregiver burden is associated with both the presence and severity of NPS, including agitation, irritability, anxiety, apathy, sleep disturbances, and other clinically disruptive symptoms (12–14). However, NPS frequently co-occur and may be correlated with one another, making it difficult to determine whether caregiver burden is more consistently associated with the cumulative burden of multiple symptoms or with particular symptom domains. Clinically, this distinction may inform whether assessment should emphasize overall NPS burden, specific high-impact symptoms, or both (12, 14).

Despite growing evidence, several important gaps remain. Few studies, however, have examined multiple global and domain-specific NPI measures within the same cohort to determine whether caregiver burden is more consistently associated with cumulative symptom load or with individual symptom domains. Evidence from Chinese family-care settings also remains comparatively limited, particularly among patient–caregiver dyads spanning MCI and multiple cognitive-disorder etiologies. In China, family members remain an important source of care for individuals with dementia, and caregiver burden is influenced by patient, caregiver, and social factors, including neuropsychiatric symptoms, care time, economic stress, health status, and social support (10). In addition, the functional form of the association between overall NPS burden and caregiver burden remains incompletely characterized. Moreover, it remains unclear whether the frequency and severity components of NPS contribute similarly or interact in their associations with caregiver burden (15).

Accordingly, the present study examined the associations of NPI total score, symptom count, caregiver-distress total score, and individual NPI domain scores with caregiver burden in a Chinese cohort spanning MCI and multiple cognitive-disorder etiologies. We additionally evaluated symptom co-occurrence, potential nonlinearity, and domain-specific frequency, severity, and within-domain frequency-by-severity interactions, while adjusting for patient clinical and functional characteristics and caregiver sociodemographic and caregiving-context factors. By examining these measures within the same analytical framework, the study aimed to evaluate whether caregiver burden is more consistently associated with cumulative and co-occurring NPS burden than with conditional domain-specific associations. Clinically, this distinction may help inform the balance between global NPS assessment for identifying families experiencing greater overall burden and domain-level assessment for understanding specific caregiving difficulties and supporting individualized management.

## 2 Materials and methods

### 2.1 Study design and participants

This single-center, cross-sectional study included patient–caregiver dyads consecutively recruited from the Neurodegenerative and Cognitive Disorders Unit of Xuanwu Hospital, Capital Medical University, between June 2022 and June 2025.

Patients were evaluated and diagnosed by neurologists specializing in cognitive disorders based on clinical history, neurological examination, neuropsychological assessment, functional evaluation, and available neuroimaging or laboratory information. Diagnoses were made according to established international criteria. Mild cognitive impairment (MCI) was diagnosed according to Petersen criteria (16), Alzheimer’s disease dementia according to the National Institute on Aging–Alzheimer’s Association criteria (17), vascular dementia or vascular cognitive disorder according to VASCOG and/or NINDS-AIREN criteria (18), dementia with Lewy bodies according to the 2017 Dementia with Lewy Bodies Consortium criteria (19), and frontotemporal dementia/frontotemporal lobar degeneration according to international consensus criteria (20).

The inclusion criteria were as follows: 1) patients had a clinical diagnosis of cognitive impairment, including MCI, Alzheimer’s disease, vascular dementia, dementia with Lewy bodies, frontotemporal lobar degeneration, mixed dementia, or other cognitive disorders, diagnosed by a neurologist according to established diagnostic criteria; 2) caregivers were family members who lived with the patient or provided regular daily care; 3) caregivers were able to understand the questionnaire items and provide their own responses in Chinese; and 4) caregivers provided written informed consent to participate.

The exclusion criteria were as follows: 1) paid professional caregiving rather than family caregiving; 2) inability of the caregiver to understand the questionnaire items or provide their own responses; and 3) patients were not living at home.

The study was approved by the Ethics Committee of Xuanwu Hospital, Capital Medical University (approval number: 2021047). Written informed consent was obtained from all participating caregivers and from patients or their legally authorized representatives when appropriate. The study was conducted in accordance with the Declaration of Helsinki.

### 2.2 Measures

Patient and caregiver characteristics were collected using a structured questionnaire administered by trained clinical personnel. For caregivers who were unable to read the questionnaires, study staff read each item aloud and recorded the responses provided by the caregivers themselves. Patient variables included age, sex, diagnosis, education, duration since cognitive-symptom onset, global cognition, and activities of daily living. The approximate duration since cognitive-symptom onset was derived from the source dataset as 2025 minus the recorded year of cognitive-symptom onset. Caregiver variables included age, sex, relationship to the patient (spouse, adult child, or other family member), education level, co-residence status, and caregiving duration. Co-residence was defined according to whether the caregiver lived with the patient, and caregiving duration was categorized as <6 months or ≥6 months.

Neuropsychiatric symptoms were assessed using the Neuropsychiatric Inventory (NPI) (21), a widely used informant-based instrument for assessing behavioral and psychological symptoms in dementia and related cognitive disorders. The NPI covers 12 symptom domains: delusions, hallucinations, agitation/aggression, depression/dysphoria, anxiety, elation/euphoria, apathy/indifference, disinhibition, irritability/lability, aberrant motor behavior, nighttime behaviors, and appetite/eating changes. Symptoms were assessed over the preceding 4 weeks. Frequency was rated from 1 (occasionally, less than once per week) to 4 (very frequently, once or more per day or continuously), and severity was rated from 1 (mild) to 3 (severe). Caregiver distress was rated from 0 (not distressing) to 5 (extremely or very severely distressing). For each domain, the domain score was calculated as frequency × severity (range, 0–12), with absent symptoms coded as 0. The NPI total score was calculated as the sum of the 12 domain scores (range, 0–144). NPI symptom count was defined as the number of domains with a positive domain score (range, 0–12), and the total caregiver-distress score was calculated as the sum of distress ratings across the 12 domains (range, 0–60). Binary indicators of symptom presence were used for prevalence estimates, symptom counts, and co-occurrence analyses. Any neuropsychiatric symptom was considered present when at least one NPI domain was positive during the preceding 4 weeks. Higher scores indicated more severe neuropsychiatric symptoms or greater symptom-related caregiver distress. The Chinese version of the NPI has demonstrated acceptable internal consistency, with a reported Cronbach’s α of 0.69 (22).

Caregiver burden was assessed using the Zarit Burden Interview (ZBI) (23). The ZBI comprises 22 items rated from 0 (never) to 4 (almost always), yielding a total score ranging from 0 to 88. Higher scores indicate greater perceived caregiver burden. The Chinese version has demonstrated good reliability and validity, with a reported Cronbach’s α of 0.875 (24). For descriptive purposes, ZBI total scores were categorized as little or no burden (0–20), mild burden (21–40), moderate burden (41–60), and severe burden (61–88). The ZBI total score was retained as a continuous outcome in the regression analyses.

Global cognitive function was assessed using the Mini-Mental State Examination (MMSE) (25) and the Montreal Cognitive Assessment (MoCA) (26). The MMSE assesses orientation, memory, attention, calculation, language, and visuospatial ability, with total scores ranging from 0 to 30; lower scores indicate poorer global cognitive function. The MoCA was used as an additional measure of global cognitive function.

Basic activities of daily living were measured using the Barthel Index (BI) (27). The BI assesses functional independence in basic daily activities, with lower scores indicating greater functional dependence. In this study, BI scores of 0–20 indicated total dependence, 21–60 severe dependence, 61–90 moderate dependence, 91–99 slight dependence, and 100 independence in basic activities of daily living.

### 2.3 Statistical analysis

Statistical analyses were performed using Python with NumPy, SciPy, statsmodels, and patsy. Descriptive statistics, Spearman correlation analyses, Mann–Whitney U tests, and preliminary data checks were conducted in SPSS. Multiple imputation, multivariable linear regression with HC3 heteroscedasticity-consistent standard errors, Rubin’s-rule pooling, nonlinearity testing, and Benjamini–Hochberg false discovery rate correction were performed in Python using predefined scripts based on pandas, statsmodels, and scipy.

Diagnostic category and caregiver relationship were treated as categorical variables. Co-residence status was coded as a binary variable (yes/no). Caregiving duration was dichotomized as <6 months versus ≥6 months in adjusted models. Educational level was modeled as an ordinal variable. Duration since cognitive-symptom onset was modeled as a continuous variable in all adjusted analyses.

The three global NPI indicators including NPI total score, NPI symptom count, and NPI caregiver-distress total score were examined in separate adjusted models using the same covariate set. For domain-level analyses, the 12 continuous NPI domain scores, each defined as the frequency × severity product (range, 0–12), were entered simultaneously into a single adjusted model. Multicollinearity among NPI domains was evaluated using variance inflation factors calculated from these 12 continuous domain scores. Binary symptom-presence indicators were not included as predictors in this simultaneous domain model and were used only for prevalence, symptom-count, and co-occurrence analyses.

To further examine symptom co-occurrence, separate adjusted models were fitted for each of the 12 NPI domains. In each model, the index-domain score and the number of other positive NPI domains were entered simultaneously, with the index domain excluded from the co-occurrence count. The same covariate set was used in all models, and P values for the 12 index-domain effects were adjusted using the Benjamini–Hochberg false discovery rate procedure.

Domain-level frequency and severity analyses were conducted within the same multiple-imputation framework as the primary adjusted analyses (N = 418; m = 50). In the frequency model, the frequency ratings of all 12 NPI domains were entered simultaneously; an analogous model included the 12 severity ratings simultaneously. We then examined within-domain frequency-by-severity interactions in separate models for each NPI domain. For each interaction model, the domain-specific frequency and severity ratings were mean-centered before their product term was constructed, and both main effects and the interaction term were retained. All models used the same covariate set as the primary domain-level model. HC3 heteroscedasticity-consistent covariance estimates were obtained within each imputed dataset, and regression coefficients and variances were pooled across the 50 imputations using Rubin’s rules. Benjamini–Hochberg FDR correction was applied separately across the 12 frequency coefficients, the 12 severity coefficients, and the 12 interaction terms.

Of the 418 patient–caregiver dyads, 403 (96.4%) had complete observed ZBI data. During data-quality review, non-integer values inconsistent with the permitted ZBI item response range of 0–4 were identified in 15 dyads and were treated as missing before the final analyses. Missing ZBI outcome scores for these 15 dyads were handled using multiple imputation with predictive mean matching, with 50 imputations generated. Regression models were fitted separately in each completed dataset, and coefficient estimates and variances were combined using Rubin’s rules. Complete-case analyses among the 403 dyads with observed ZBI scores were performed as sensitivity analyses.

Clinically and logically plausible values within the permitted scale ranges were retained, and no participant was excluded solely because of an extreme NPI or ZBI score. Influential observations were evaluated using Cook’s distance. Because several continuous variables showed skewed distributions, Spearman rank correlations were used for unadjusted analyses. NPI and ZBI scores were retained on their original scales to preserve clinical interpretability; no logarithmic, square-root, or other transformations were applied. HC3 heteroscedasticity-consistent standard errors were used in the adjusted linear regression models. Model diagnostics included visual examination of residual patterns, assessment of heteroscedasticity using the Breusch–Pagan test, multicollinearity using variance inflation factors, and influential observations using Cook’s distance.

For domain-level analyses, *P* values were adjusted using the Benjamini–Hochberg false discovery rate procedure across 12 tests. Potential nonlinearity in the association between NPI total score and ZBI score was examined using both a quadratic term and a natural cubic restricted cubic spline model. Additional sensitivity analyses replaced MMSE with MoCA, repeated the analyses in complete cases, and excluded influential observations. All statistical tests were two-sided, and *P* < 0.05 was considered statistically significant. All analyses were conducted using predefined scripts to support reproducibility.

## 3 Results

### 3.1 Participant characteristics

The study included 418 patient–caregiver dyads with cognitive impairment. Patients had a mean age of 66.2 ± 9.1 years, 166 (39.7%) were aged <65 years, 185 (44.3%) were aged 65–74 years, and 67 (16.0%) were aged ≥75 years, and 233 (55.7%) were female. The most common diagnosis was Alzheimer’s disease (195, 46.7%), followed by MCI (109, 26.1%). The mean approximate duration since cognitive-symptom onset was 5.9 years (SD, 1.9; range, 1–11 years). Mean MMSE and MoCA scores were 18.6 ± 7.8 and 14.1 ± 7.9, respectively. Caregivers had a mean age of 56.7 ± 15.1 years; 250 (59.8%) were female, and most were spouses or adult children of the patients (**Table 1**).

**Table 1:** Demographic and clinical characteristics of patients and caregivers (n = 418).

Patient characteristics	
Age (years), mean ± SD	66.2 ± 9.1
Age <65 years, n (%)	166 (39.7)
Age 65–74 years, n (%)	185 (44.3)
Age ≥75 years, n (%)	67 (16.0)
Female, n (%)	233 (55.7)
Diagnosis, n (%)	
Mild cognitive impairment (MCI)	109 (26.1)
Alzheimer’s disease (AD)	195 (46.7)
Vascular dementia (VaD)	38 (9.1)
Dementia with Lewy bodies (DLB)	19 (4.5)
Frontotemporal dementia/frontotemporal lobar degeneration	20 (4.8)
Mixed dementia	20 (4.8)
Other cognitive disorders	17 (4.1)
Patient education level, n (%)	
Illiterate	19 (4.5)
Primary school	48 (11.5)
Junior high school	96 (23.0)
High school/vocational school	119 (28.5)
College/university	122 (29.2)
Postgraduate	14 (3.3)
MMSE, mean ± SD	18.6 ± 7.8
MoCA, mean ± SD	14.1 ± 7.9
Duration since cognitive-symptom onset, years	5.9 ± 1.9
BI, mean ± SD	67.0 ± 20.5
NPI total score, median (IQR)	3.0 (0.0–12.0)
NPI symptom count, median (IQR)	1.0 (0.0–3.0)
NPI caregiver distress score, mean ± SD	2.9 ± 5.2
ZBI, median (IQR)	14.0 (6.0-26.0)
Caregiver characteristics	
Age (years), mean ± SD	56.7 ± 15.1
Female, n (%)	250 (59.8)
Relationship to patient, n (%)	
Spouse	265 (63.4)
Child	140 (33.5)
Other family member	13 (3.1)
Caregiver education level, n (%)	
Illiterate	7 (1.7)
Primary school	7 (1.7)
Junior high school	43 (10.3)
High school/vocational school	82 (19.6)
College/university	228 (54.5)
Postgraduate	51 (12.2)
Co-residence with the patient, yes, n (%)	388 (92.8)
Duration of caregiving	
<6 months	46 (11.0)
≥6 months	372 (89.0)

Values are presented as mean ± SD, median (*IQR*), or *n* (%), as appropriate. ADL, activities of daily living; BI, Barthel Index; MMSE, Mini-Mental State Examination; MoCA, Montreal Cognitive Assessment; NPI, Neuropsychiatric Inventory; ZBI, Zarit Burden Interview. Descriptive statistics for the ZBI were calculated among the 403 participants with complete observed ZBI data.

Among the 403 dyads with complete observed ZBI data, the mean ZBI total score was 17.2 ± 14.7, with a median of 14.0 (IQR, 6.0–26.0). Of these caregivers, 261 (64.8%) had little or no burden, 116 (28.8%) had mild burden, 21 (5.2%) had moderate burden, and 5 (1.2%) had severe burden.

### 3.2 Prevalence and distribution of neuropsychiatric symptoms of cognitive impairment

In total, 251 patients (60.0%; 95% CI, 55.3%–64.6%) had at least one NPS. The five most prevalent domains were apathy/indifference (30.4%), depression/dysphoria (25.1%), anxiety (23.0%), nighttime behaviors (21.1%), and irritability/lability (18.7%) (**Table 2**). The median number of positive NPI domains was 1.0 (IQR, 0.0–3.0). Overall, 167 patients (40.0%) had no NPS, whereas 141 patients (33.7%) had three or more positive NPI domains (**Table 3**).

**Table 2:** Prevalence of specific neuropsychiatric symptoms (n = 418).

NPI domain	n/N	%	95% CI
Delusions	54/418	12.9	10.0–16.5
Hallucinations	42/418	10.0	7.5–13.3
Agitation/aggression	50/418	12.0	9.2–15.4
Depression/dysphoria	105/418	25.1	21.2–29.5
Anxiety	96/418	23.0	19.2–27.2
Euphoria/elation	25/418	6.0	4.1–8.7
Apathy/indifference	127/418	30.4	26.2–35.0
Disinhibition	42/418	10.0	7.5–13.3
Irritability/lability	78/418	18.7	15.2–22.7
Aberrant motor behavior	62/418	14.8	11.7–18.6
Nighttime behaviors	88/418	21.1	17.4–25.2
Appetite/eating changes	48/418	11.5	8.8–14.9

Symptom presence was defined as a positive NPI domain score (>0); absent symptoms were coded as 0.

**Table 3:** Distribution of the number of neuropsychiatric symptom domains per patient.

Number of NPS domains	n	%	Cumulative %
0	167	40.0	40.0
1	72	17.2	57.2
2	38	9.1	66.3
3	57	13.6	79.9
4	25	6.0	85.9
5	24	5.7	91.6
6	13	3.1	94.7
7	3	0.7	95.5
8	7	1.7	97.1
9	3	0.7	97.8
10	5	1.2	99.0
11	2	0.5	99.5
12	2	0.5	100.0

### 3.3 Associations between neuropsychiatric symptoms and caregiver burden

Among the 403 dyads with complete observed ZBI data, caregivers of patients with at least one NPS had higher ZBI scores than caregivers of patients without NPS (median, 15.0 [IQR, 8.0–29.0] vs. 10.0 [IQR, 3.0–21.0]; Mann–Whitney U test, P < 0.001).

Spearman correlation analyses among the 403 dyads with complete observed ZBI data showed that ZBI total score was positively correlated with NPI total score (ρ = 0.308, P < 0.001), NPI symptom count (ρ = 0.313, P < 0.001), and NPI caregiver-distress total score (ρ = 0.336, P < 0.001). BI, MMSE, and MoCA scores were not significantly correlated with ZBI total score. All 12 NPI domain scores showed statistically significant positive bivariate correlations with ZBI total score, although the effect sizes were small to moderate (**Table 4**).

**Table 4:** Spearman correlations with caregiver burden measured by ZBI total score among participants with complete observed ZBI data (n = 403).

Variable	n	Spearman *ρ*	*P* value
NPI total score	403	0.308	<0.001
NPI symptom count	403	0.313	<0.001
NPI caregiver distress score	403	0.336	<0.001
MMSE	403	-0.055	0.275
BI score	403	-0.074	0.139
MoCA	403	-0.047	0.346
Delusions score	403	0.240	<0.001
Hallucinations score	403	0.179	<0.001
Agitation/aggression score	403	0.132	0.008
Depression/dysphoria score	403	0.158	0.001
Anxiety score	403	0.233	<0.001
Euphoria/elation score	403	0.125	0.012
Apathy/indifference score	403	0.238	<0.001
Disinhibition score	403	0.204	<0.001
Irritability/lability score	403	0.107	0.032
Aberrant motor behavior score	403	0.277	<0.001
Nighttime behaviors score	403	0.201	<0.001
Appetite/eating changes score	403	0.128	0.010

ZBI, Zarit Burden Interview; BI, Barthel Index; MMSE, Mini-Mental State Examination; MoCA, Montreal Cognitive Assessment; NPI, Neuropsychiatric Inventory.

### 3.4 Global NPI models and nonlinearity

In the multiple-imputation analysis (m = 50) including all 418 dyads, separate fully adjusted models showed positive associations of caregiver burden with NPI total score (B = 0.357; 95% CI, 0.236–0.478; standardized β = 0.339; P < 0.001), NPI symptom count (B = 2.048; 95% CI, 1.368–2.729; standardized β = 0.340; P < 0.001), and NPI caregiver-distress total score (B = 1.018; 95% CI, 0.640–1.396; standardized β = 0.357; P < 0.001). A 10-point increase in NPI total score corresponded to an adjusted 3.6-point higher ZBI total score. The mean R² and adjusted R² of the NPI total-score model across the 50 completed datasets were 0.143 and 0.099, respectively.

The mean-centered quadratic term was negative and statistically significant (B = −0.00461; 95% CI, −0.00895 to −0.00026; P = 0.038), suggesting modest concave curvature in the association. However, the nonlinear component of the restricted cubic restricted cubic spline model was not statistically significant (P = 0.245). Furthermore, after excluding six participants with NPI total scores ≥60, neither the quadratic term (P = 0.499) nor the restricted cubic spline nonlinear component (P = 0.952) was significant. Thus, evidence for nonlinearity was inconsistent across analyses, and the linear model was retained as the primary summary (**Figure 1**).

**Figure 1:**
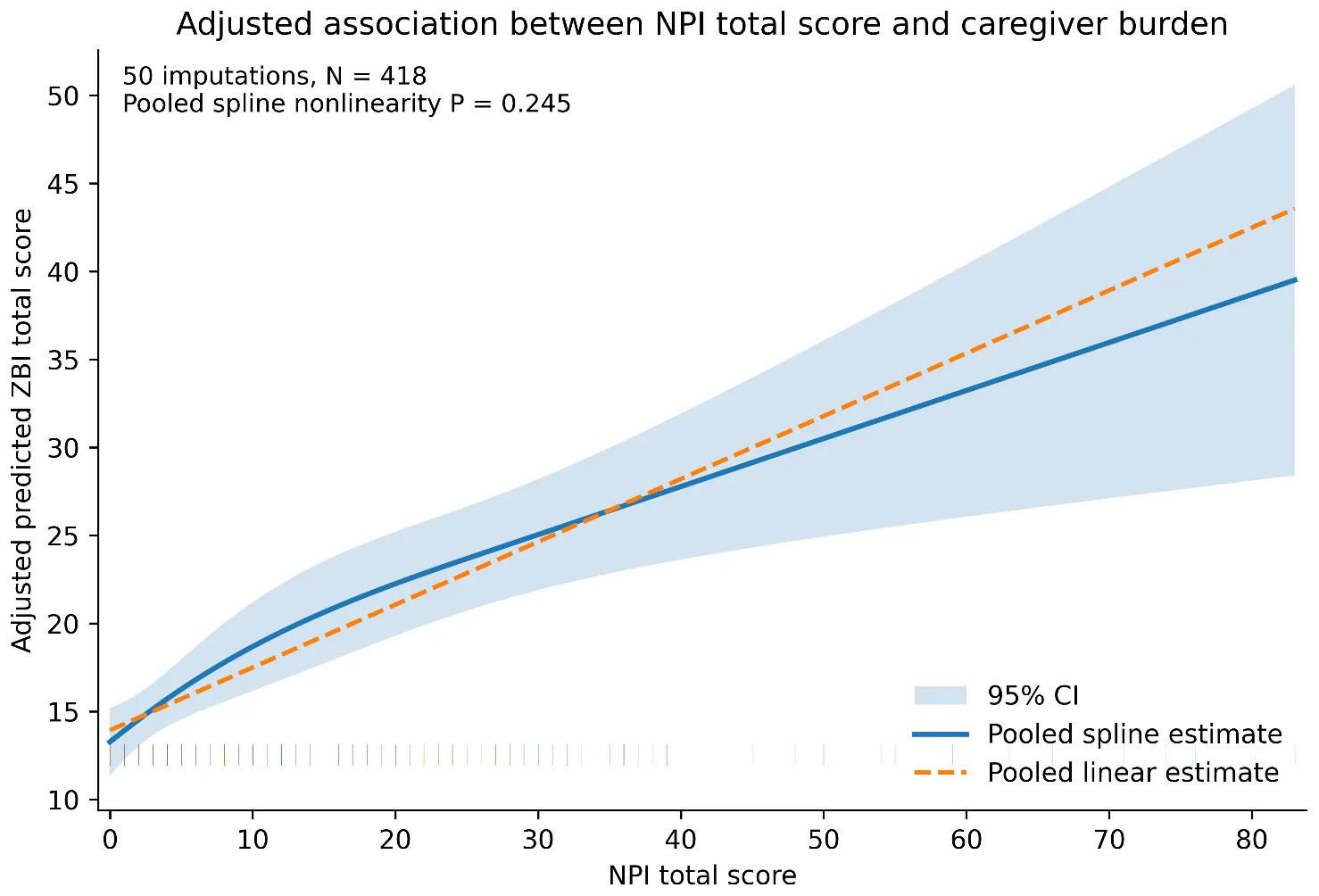
Adjusted pooled association between NPI total score and ZBI total score. The solid curve and shaded 95% confidence interval represent the pooled restricted cubic restricted cubic spline estimate across 50 imputed datasets (N = 418), and the dashed line represents the pooled linear estimate. Models were adjusted for patient age, sex, diagnosis, education, approximate duration since cognitive-symptom onset, MMSE, Barthel Index, caregiver age, sex, education, relationship, co-residence status, and caregiving duration. The pooled test for the nonlinear restricted cubic spline component was not statistically significant (P = 0.245).

### 3.5 Symptom co-occurrence analysis

After adjustment for the number of other positive NPI domains, several index-domain effects were nominally significant, but none remained statistically significant after Benjamini–Hochberg FDR correction (minimum q = 0.122). In contrast, the number of other positive domains was consistently associated with higher ZBI scores across all index-domain models. These findings suggest that caregiver burden may be more consistently related to the accumulation of co-occurring NPS than to any single symptom domain.

### 3.6 Domain-level frequency, severity, and interaction analyses

In the multiple-imputation analyses including all 418 dyads, the simultaneous frequency model showed nominal positive associations for delusions (B = 2.083; P = 0.046), anxiety (B = 2.334; P = 0.045), apathy/indifference (B = 1.991; P = 0.008), and disinhibition (B = 3.456; P = 0.027), but none remained significant after FDR correction (minimum q = 0.090). In the simultaneous severity model, anxiety (B = 2.730; P = 0.039) and nighttime behaviors (B = 2.293; P = 0.032) were nominally associated with ZBI total score, but no domain survived FDR correction (minimum q = 0.232). In the within-domain interaction models, the frequency-by-severity terms for depression/dysphoria (B = −2.171; P = 0.029) and apathy/indifference (B = −2.431; 95% CI, −4.098 to −0.765; P = 0.004) were nominally significant. Neither remained significant after FDR correction; the apathy/indifference interaction was close to, but did not cross, the prespecified FDR threshold (q = 0.051). Thus, these analyses did not provide FDR-corrected evidence that caregiver burden was disproportionately greater when a specific symptom domain was both more frequent and more severe. Full estimates are provided in **Supplementary Table S1**.

### 3.7 Associations between NPS and cognitive function

NPI total score was not significantly correlated with MMSE score (ρ = −0.038, P = 0.433) or MoCA score (ρ = −0.035, P = 0.470). These findings indicate that global NPS severity was not closely aligned with global cognitive test performance in this sample.

### 3.8 Domain-level multivariable regression analysis

When all 12 NPI domain scores were entered simultaneously with the fully adjusted covariate set in the multiple-imputation analysis (N = 418, m = 50), no individual domain was statistically significant either before or after Benjamini–Hochberg FDR correction (minimum FDR q = 0.301; **Table 5**). Because these coefficients are conditional estimates obtained while holding the other correlated NPI domains constant, negative coefficients may arise from shared variance and mutual adjustment among domains. The nonsignificant negative coefficients and their confidence intervals spanning zero should therefore not be interpreted as indicating protective associations. In the complete-case diagnostic model, multicollinearity was low (maximum VIF = 1.76), the Breusch–Pagan test did not indicate heteroscedasticity (P = 0.654), and the maximum Cook’s distance was 0.065.

**Table 5:** Fully adjusted linear regression models of NPI indicators and ZBI total score.

Model	Predictor	B	95% CI	β	P	q
Model 1: NPI total score	NPI total score	0.357	0.236–0.478	0.339	<0.001	–
Model 2: individual domains	Delusions	0.725	−0.203–1.653	0.104	0.125	0.301
	Hallucinations	−0.202	−1.144–0.740	−0.025	0.674	0.809
	Agitation/aggression	0.715	−0.212–1.643	0.080	0.131	0.301
	Depression/dysphoria	−0.592	−1.499–0.314	−0.101	0.200	0.301
	Anxiety	0.815	−0.204–1.833	0.136	0.117	0.301
	Euphoria/elation	0.750	−1.002–2.503	0.055	0.401	0.535
	Apathy/indifference	0.442	−0.066–0.950	0.101	0.088	0.301
	Disinhibition	1.277	−0.594–3.148	0.114	0.181	0.301
	Irritability/lability	−0.056	−1.142–1.030	−0.008	0.919	0.919
	Aberrant motor behavior	0.586	−0.249–1.421	0.105	0.169	0.301
	Nighttime behaviors	0.547	−0.117–1.211	0.113	0.106	0.301
	Appetite/eating changes	−0.043	−0.883–0.797	−0.006	0.919	0.919

N = 418 patient-caregiver dyads; m = 50 imputations. B, unstandardized regression coefficient; beta, standardized regression coefficient; CI, confidence interval; q, Benjamini-Hochberg false-discovery-rate-adjusted P value across the 12 NPI domains in Model 2. Regression coefficients and HC3 heteroscedasticity-consistent covariance estimates were obtained within each imputed dataset, and coefficients and variances were pooled using Rubin's rules. All 12 continuous NPI domain scores were entered simultaneously in Model 2. Both models adjusted for patient age, sex, diagnosis, education, approximate duration since cognitive-symptom onset, MMSE, Barthel Index, caregiver age, sex, education, relationship, co-residence status, and caregiving duration.

In the domain-level model, all 12 continuous NPI domain scores were entered simultaneously. Each domain score was calculated as frequency × severity (range, 0–12), with absent symptoms coded as 0. Binary symptom-presence indicators were not used as predictors in this model. VIFs for multicollinearity assessment were calculated using these 12 continuous domain scores.

### 3.9 Sensitivity analyses

Sensitivity analyses yielded results consistent with the primary analysis. In the complete-case analysis of 403 dyads, the association between NPI total score and ZBI total score remained significant (B = 0.356; 95% CI, 0.235–0.478; standardized β = 0.341; P < 0.001). Similar results were obtained when MMSE was replaced by MoCA in the multiple-imputation model (B = 0.357; 95% CI, 0.236–0.479; standardized β = 0.339; P < 0.001). After excluding 25 observations exceeding the prespecified Cook’s distance threshold of 4/n, the association also remained significant (N = 393; B = 0.349; 95% CI, 0.270–0.428; standardized β = 0.384; P < 0.001).

## 4 Discussion

In this cross-sectional study of 418 patient–caregiver dyads, NPS were common among individuals with cognitive impairment, with 60.0% having at least one positive NPI domain. The most frequently observed symptoms were apathy/indifference, depression/dysphoria, anxiety, nighttime behaviors, and irritability/lability. The principal finding was that caregiver burden was consistently associated with cumulative NPS measures, including NPI total score, symptom count, and caregiver-distress total score, whereas no individual NPI domain remained statistically significant in the simultaneous domain model after FDR correction. The association between NPI total score and ZBI total score remained consistent across multiple-imputation and sensitivity analyses.

The prevalence and distribution of NPS in this study are broadly consistent with recent evidence showing that behavioral and psychological symptoms are common across the continuum of cognitive impairment. Kwon and Lee (28) reported that apathy, depression, anxiety, irritability, agitation/aggression, sleep disorders, and eating disorders were among the relatively common BPSD characteristics in community-dwelling patients with dementia. Kanemoto et al. (29) further showed that BPSD factors were clinically relevant to caregiver burden in dementia with Lewy bodies, while Lu et al. (30) reported that BPSD profiles and caregiver distress differed between community-dwelling and nursing-home residents with dementia. Similarly, Iravani et al. (15) found that NPS were associated with caregiver burden in patients with Alzheimer’s disease, with apathy being one of the most common symptoms. These findings, together with our results, suggest that NPS are not peripheral manifestations of cognitive impairment, but clinically meaningful symptoms that vary across disease subtypes, stages, and care environments.

Apathy was the most common neuropsychiatric symptom domain in this cohort. Although apathy may be less overtly disruptive than agitation or psychosis, previous studies have suggested that it can increase the need for prompting, supervision, and assistance with daily activities (12, 31). Depression, anxiety, and nighttime behaviors were also common and may interfere with emotional interaction, sleep, and daily caregiving routines (12). However, none of these individual domains remained significant after simultaneous adjustment and FDR correction in the present study; therefore, the domain-level findings should be interpreted as descriptive and exploratory rather than as evidence that any single symptom independently determines caregiver burden.

Separating the frequency and severity components at the domain level did not materially change the overall interpretation. Several frequency and severity coefficients were nominally associated with caregiver burden, but none survived FDR correction. Likewise, no within-domain frequency-by-severity interaction met the prespecified FDR threshold. The apathy/indifference interaction was the closest (q = 0.051) and had a negative coefficient. Because no interaction survived FDR correction, this result should be regarded as an exploratory signal rather than evidence of a domain-specific moderating effect. The negative coefficient should not be interpreted as showing that greater apathy severity reduces caregiver burden; it indicates only that the frequency–burden association was not simply additive across severity levels. Replication in independent cohorts is needed.

These findings are consistent with previous studies suggesting that neuropsychiatric symptom clusters are variably associated with caregiver distress across disease stages and care settings (13, 14, 29). NPI domains frequently co-occur, and this overlap may partly account for the attenuation of individual domain effects in multivariable models (14).

One important feature of this study is that we examined both global NPI indicators and individual NPI domains. NPI total score, symptom count, and caregiver-distress score each showed consistent associations with ZBI score, whereas no individual domain remained statistically significant when all 12 domains were entered simultaneously and the results were corrected for multiple testing. This does not imply that individual symptoms are clinically unimportant. Rather, the attenuation of domain-specific coefficients may reflect symptom co-occurrence, shared variance across domains, and limited precision for less prevalent symptoms (14). The negative coefficients observed for some domains in the simultaneous model should also be interpreted cautiously. These coefficients represent conditional associations after adjustment for the other correlated NPI domains and therefore differ from the corresponding unadjusted associations. Shared variance among domains can attenuate coefficients or change their signs after mutual adjustment. Notably, all 12 domain scores were positively correlated with ZBI total score in the unadjusted analyses, whereas the negative coefficients in the simultaneous model were nonsignificant and their confidence intervals included zero. Thus, these estimates should not be interpreted as suggesting that greater symptom severity is associated with lower caregiver burden.

These findings are compatible with the interpretation that perceived caregiver burden may be more consistently related to the accumulation and co-occurrence of neuropsychiatric symptoms than to one isolated symptom (12, 14). García-Martín et al. reported associations of caregiver burden with both the presence and intensity of NPS (13), whereas van den Kieboom et al. found that overall NPS were associated with family caregiver burden but that differences among symptom clusters were not conclusive (14). Global NPI measures may therefore be useful for identifying families experiencing greater overall burden, while domain-level assessment remains relevant for understanding specific management difficulties and planning individualized support.

A recent systematic review and meta-regression reported a positive association between neuropsychiatric symptoms and caregiver distress measured by the NPI-D; however, the meta-regression of NPI total score and ZBI caregiver burden was not statistically significant. That study did not evaluate within-domain frequency-by-severity interactions, so the present component analyses should remain exploratory (32).

Global cognitive measures (MMSE/MoCA) and functional status (BI) were not significantly associated with caregiver burden in univariate analyses. This suggests that behavioral symptoms capture aspects of caregiving difficulty that are not reflected in standard cognitive or functional assessments.

Taken together, these findings support considering cumulative neuropsychiatric symptom burden alongside domain-level assessment when evaluating caregiver experience in patients with cognitive impairment (13).

Practical implications: Caregiver-support strategies may need to address both overall symptom burden and symptom patterns that interfere with daily care. For nighttime behaviors, individualized non-pharmacological approaches, including activity-based, environmental, and caregiver-focused strategies, may be considered, although evidence for specific interventions remains limited (33). For anxiety, structured cognitive and physical activities and other non-pharmacological approaches may be considered (34). Apathy may warrant individualized therapeutic activities and caregiver guidance (31). Respite services may also be considered as part of broader caregiver support, although evidence regarding their effects is heterogeneous (35).

## 5 Limitations

This study has several limitations. First, the cross-sectional design precludes causal inference; longitudinal studies are needed to determine whether changes in NPS predict subsequent changes in caregiver burden or whether these associations are bidirectional. Second, participants were recruited from a single tertiary specialized cognitive-disorders inpatient unit, and the relatively young age distribution of the cohort may limit generalizability to community-based, primary care, or predominantly older, late-onset populations. Third, the NPI assesses symptoms over the preceding 4 weeks and both NPI and ZBI ratings were based on caregiver report, raising the possibility of shared-method variance and potential underestimation of chronic or fluctuating NPS exposure. Fourth, although co-residence status and caregiving duration were included in the adjusted models, more detailed caregiving information (e.g., daily caregiving hours, paid-care support, caregiver coping style) was not captured, and some ZBI data required multiple imputation; residual confounding and bias related to missing-data assumptions cannot be excluded. Fifth, duration since cognitive-symptom onset was estimated from the recorded year of onset rather than exact dates and should be interpreted as an approximate measure.

## 6 Conclusion

NPS were common among individuals with cognitive impairment and were associated with greater family caregiver burden. Overall NPI severity, symptom count, and caregiver distress were consistently associated with ZBI score after adjustment for patient and caregiver characteristics. In contrast, no individual symptom domain remained statistically significant in the simultaneous domain model after FDR correction. These findings suggest the potential value of assessing overall neuropsychiatric symptom burden while remaining attentive to individual symptoms that may pose specific caregiving challenges.

## Data Availability

The raw data supporting the conclusions of this article will be made available by the authors, without undue reservation.

## References

[1] GBD 2019 Dementia Forecasting Collaborators . Estimation of the global prevalence of dementia in 2019 and forecasted prevalence in 2050: an analysis for the Global Burden of Disease Study 2019. Lancet Public Health. (2022) 7:e105–e25. doi: 10.1016/S2468-2667(21)00249-8 PMC8810394 34998485

[2] Jia L Du Y Chu L Zhang Z Li F Lyu D . Prevalence, risk factors, and management of dementia and mild cognitive impairment in adults aged 60 years or older in China: a cross-sectional study. Lancet Public Health. (2020) 5:e661–e71. doi: 10.1016/s2468-2667(20)30185-7 33271079

[3] Petersen RC Lopez O Armstrong MJ Getchius TSD Ganguli M Gloss D . Practice guideline update summary: Mild cognitive impairment: report of the Guideline Development, Dissemination, and Implementation Subcommittee of the American Academy of Neurology. Neurology. (2018) 90:126–35. doi: 10.1212/wnl.0000000000006042 29282327 PMC5772157

[4] Pozzi FE Calì L Ferrarese C Appollonio I Tremolizzo L . Assessing behavioral and psychological symptoms of dementia: a comprehensive review of current options and future perspectives. Front Dementia. (2023) 2:1226060. doi: 10.3389/frdem.2023.1226060 39082001 PMC11285590

[5] Fan Z Wang L Zhang H Lv X Tu L Zhang M . Apathy as a risky neuropsychiatric syndrome of progression from normal aging to mild cognitive impairment and dementia: a systematic review and meta-analysis. Front Psychiatry. (2021) 12:792168. doi: 10.3389/fpsyt.2021.792168 34987434 PMC8721876

[6] Fresnais D Humble MB Bejerot S Meehan AD Fure B . Apathy as a Predictor for Conversion From Mild Cognitive Impairment to Dementia: A Systematic Review and Meta-Analysis of Longitudinal Studies. J Geriatr Psychiatry Neurol. (2023) 36(1):3–17. doi: 10.1177/08919887221093361 35446723 PMC9755689

[7] Tu JY Jin G Chen JH Chen YC . Caregiver burden and dementia: a systematic review of self-report instruments. J Alzheimer's Dis. (2022) 86:1527–43. doi: 10.3233/jad-215082 35253744

[8] Strini V Prendin A Cerrone V Schiavolin R de Barbieri I Andretta V . Scale of assessment of caregiver care burden of people with dementia: a systematic review of literature. Transl Med UniSa. (2023) 25:38–49. doi: 10.37825/2239-9747.1044 38343418 PMC10851772

[9] Oliveira D da Mata FAF Aubeeluck A . Quality of life of family carers of people living with dementia: review of systematic reviews of observational and intervention studies. Br Med Bull. (2024) 149:1–12. doi: 10.1093/bmb/ldad029 38050333

[10] Wang L Zhou Y Fang X Qu G . Care burden on family caregivers of patients with dementia and affecting factors in China: a systematic review. Front Psychiatry. (2022) 13:1004552. doi: 10.3389/fpsyt.2022.1004552 36545040 PMC9760850

[11] Tampi RR Jeste DV . Dementia is more than memory loss: neuropsychiatric symptoms of dementia and their nonpharmacological and pharmacological management. Am J Psychiatry. (2022) 179:528–43. doi: 10.1176/appi.ajp.20220508 35921394

[12] Terum TM Andersen JR Rongve A Aarsland D Svendsboe EJ Testad I . The relationship of specific items on the Neuropsychiatric Inventory to caregiver burden in dementia: a systematic review. Int J Geriatric Psychiatry. (2017) 32:703–17. doi: 10.1002/gps.4704 28317166

[13] García-Martín V de Hoyos-Alonso MC Delgado-Puebla R Ariza-Cardiel G Del Cura-González I . Burden in caregivers of primary care patients with dementia: influence of neuropsychiatric symptoms according to disease stage (NeDEM project). BMC Geriatrics. (2023) 23:525. doi: 10.1016/S2468-2667(21)00249-8 PMC10463529 37644410

[14] van den Kieboom R Snaphaan L Mark R van Assen M Bongers I . The effects of neuropsychiatric symptom clusters in people with dementia on family caregiver burden. J Alzheimer's Dis. (2024) 100:1289–98. doi: 10.3233/jad-230972 38995773 PMC11380262

[15] Iravani B Abdollahi E Eslamdoust-Siahestalkhi F Soleimani R . Neuropsychiatric symptoms of alzheimer's disease and caregiver burden. Front Neurol. (2022) 13:877143. doi: 10.3389/fneur.2022.877143 35968317 PMC9372403

[16] Petersen RC . Mild cognitive impairment as a diagnostic entity. J Intern Med. (2004) 256:183–94. doi: 10.1111/j.1365-2796.2004.01388.x 15324362

[17] McKhann GM Knopman DS Chertkow H Hyman BT Jack CR Jr. Kawas CH . The diagnosis of dementia due to Alzheimer's disease: Recommendations from the National Institute on Aging-Alzheimer's Association workgroups on diagnostic guidelines for Alzheimer's disease. Alzheimer's Dementia. (2011) 7:263–9. doi: 10.1016/j.jalz.2011.03.005 21514250 PMC3312024

[18] Sachdev P Kalaria R O'Brien J Skoog I Alladi S Black SE . Diagnostic criteria for vascular cognitive disorders: a VASCOG statement. Alzheimer Dis Associated Disord. (2014) 28:206–18. doi: 10.1097/WAD.0000000000000034 PMC4139434 24632990

[19] McKeith IG Boeve BF Dickson DW Halliday G Taylor J-P Weintraub D . Diagnosis and management of dementia with Lewy bodies: Fourth consensus report of the DLB Consortium. Neurology. (2017) 89:88–100. doi: 10.1212/wnl.0000000000004919 28592453 PMC5496518

[20] Rascovsky K Hodges JR Knopman D Mendez MF Kramer JH Neuhaus J . Sensitivity of revised diagnostic criteria for the behavioural variant of frontotemporal dementia. Brain. (2011) 134:2456–77. doi: 10.1093/brain/awr179 21810890 PMC3170532

[21] Cummings JL Mega M Gray K Rosenberg-Thompson S Carusi DA Gornbein J . The Neuropsychiatric Inventory: comprehensive assessment of psychopathology in dementia. Neurology. (1994) 44:2308–14. doi: 10.1212/WNL.44.12.2308 7991117

[22] Wang T Xiao S Li X Wang H Liu Y Fang Y . Reliability and validity of the Chinese version of the neuropsychiatric inventory in mainland China. Int J Geriatric Psychiatry. (2012) 27:539–44. doi: 10.1002/gps.2757 21769938

[23] Zarit SH Reever KE Bach-Peterson J . Relatives of the impaired elderly: correlates of feelings of burden. Gerontologist. (1980) 20:649–55. doi: 10.1093/geront/20.6.649 7203086

[24] Lu L Wang L Yang X Feng Q . Zarit Caregiver Burden Interview: development, reliability and validity of the Chinese version. Psychiatry Clin Neurosci. (2009) 63:730–4. doi: 10.1111/j.1440-1819.2009.02019.x 19781014

[25] Folstein MF Folstein SE McHugh PR . "Mini-mental state". A practical method for grading the cognitive state of patients for the clinician. J Psychiatr Res. (1975) 12:189–98. doi: 10.1016/0022-3956(75)90026-6 1202204

[26] Lu J Li D Li F Zhou A Wang F Zuo X . Montreal cognitive assessment in detecting cognitive impairment in Chinese elderly individuals: a population-based study. J Geriatric Psychiatry Neurol. (2011) 24:184–90. doi: 10.1177/0891988711422528 22228824

[27] Mahoney FI Barthel DW . Functional evaluation: the Barthel Index. Md State Med J. (1965) 14:61–5. 14258950

[28] Kwon C-Y Lee B . Prevalence of behavioral and psychological symptoms of dementia in community-dwelling dementia patients: A systematic review. Front Psychiatry. (2021) 12:741059. doi: 10.3389/fpsyt.2021.741059 34744832 PMC8566725

[29] Kanemoto H Sato S Satake Y Koizumi F Taomoto D Kanda A . Impact of behavioral and psychological symptoms on caregiver burden in patients with dementia with lewy bodies. Front Psychiatry. (2021) 12:753864. doi: 10.3389/fpsyt.2021.753864 34777057 PMC8578553

[30] Lu X Ye R Wu J Rao D Liao X . Comparing behavioral and psychological symptoms of dementia and caregiver distress caused between older adults with dementia living in the community and in nursing homes. Front Psychiatry. (2022) 13:881215. doi: 10.3389/fpsyt.2022.881215 35651819 PMC9150772

[31] Brodaty H Burns K . Nonpharmacological management of apathy in dementia: a systematic review. Am J Geriatric Psychiatry. (2012) 20:549–64. doi: 10.1097/jgp.0b013e31822be242 21860324

[32] Moura CB Porto AM Calazans LL Sena L Leite M González FR . Neuropsychiatric symptoms of dementia and caregiver burden: a systematic review and meta-regression. Dement Neuropsychol. (2026) 20:e20250369. doi: 10.1590/1980-5764-dn-2025-0369 42516128 PMC13403518

[33] Wilfling D Calo S Dichter MN Meyer G Möhler R Köpke S . Non-pharmacological interventions for sleep disturbances in people with dementia. Cochrane Database Syst Rev. (2023) CD011881. doi: 10.1002/14651858.CD011881.pub2 36594432 PMC9808594

[34] Nimmons D Aker N Burnand A Jordan KP Cooper C Davies N . Clinical effectiveness of pharmacological and non-pharmacological treatments for the management of anxiety in community dwelling people living with dementia: A systematic review and meta-analysis. Neurosci Biobehav Rev. (2024) 157:105507. doi: 10.1016/j.neubiorev.2023.105507 38097097

[35] Vandepitte S Van Den Noortgate N Putman K Verhaeghe S Verdonck C Annemans L . Effectiveness of respite care in supporting informal caregivers of persons with dementia: a systematic review. Int J Geriatric Psychiatry. (2016) 31:1277–88. doi: 10.1002/gps.4504 27245986

