# Supplementary material for: Overall and domain-specific neuropsychiatric symptoms in relation to caregiver burden among family caregivers of individuals with cognitive impairment
Source: Front Psychiatry. 2026 Sep 30;17:1937017. doi: 10.3389/fpsyt.2026.1937017 (PMC13664287; doi:10.3389/fpsyt.2026.1937017)
Supplement: Supplementary file 1 [file Table1.docx]

**Supplementary Table S1. Domain-level frequency, severity, and within-domain frequency-by-severity analyses of caregiver burden**

Analyses were conducted within the same multiple-imputation framework as the primary adjusted analyses (N = 418; m = 50), with 15 ZBI outcomes imputed using predictive mean matching. All models adjusted for patient age, sex, diagnosis, education, duration since cognitive-symptom onset, MMSE, Barthel Index, caregiver age, sex, education, relationship, co-residence, and caregiving duration. HC3 heteroscedasticity-consistent covariance estimates were obtained within each imputed dataset, and coefficients and variances were pooled across imputations using Rubin’s rules. In Panels A and B, all 12 domain-specific frequency or severity ratings were entered simultaneously. In Panel C, a separate model was fitted for each domain with mean-centered frequency and severity main effects and their product term. Benjamini–Hochberg FDR correction was applied separately within each panel across 12 tests.

**Panel A. Simultaneous 12-domain frequency model**

| **NPI domain** | **B** | **95% CI lower** | **95% CI upper** | **P value** | **FDR q** |
| --- | --- | --- | --- | --- | --- |
| Delusions | 2.083 | 0.036 | 4.131 | 0.046 | 0.138 |
| Hallucinations | −0.395 | −2.613 | 1.822 | 0.727 | 0.780 |
| Agitation/aggression | 1.028 | −0.835 | 2.892 | 0.280 | 0.604 |
| Depression/dysphoria | −0.690 | −2.734 | 1.353 | 0.508 | 0.762 |
| Anxiety | 2.334 | 0.056 | 4.613 | 0.045 | 0.138 |
| Euphoria/elation | 0.506 | −3.049 | 4.061 | 0.780 | 0.780 |
| Apathy/indifference | 1.991 | 0.531 | 3.452 | 0.008 | 0.090 |
| Disinhibition | 3.456 | 0.397 | 6.515 | 0.027 | 0.138 |
| Irritability/lability | −0.590 | −3.065 | 1.886 | 0.641 | 0.780 |
| Aberrant motor behavior | 1.100 | −0.990 | 3.189 | 0.302 | 0.604 |
| Nighttime behaviors | 0.555 | −0.837 | 1.948 | 0.434 | 0.744 |
| Appetite/eating changes | −0.323 | −2.308 | 1.662 | 0.750 | 0.780 |

**Panel B. Simultaneous 12-domain severity model**

| **NPI domain** | **B** | **95% CI lower** | **95% CI upper** | **P value** | **FDR q** |
| --- | --- | --- | --- | --- | --- |
| Delusions | 2.388 | −0.412 | 5.188 | 0.095 | 0.284 |
| Hallucinations | −1.027 | −3.845 | 1.792 | 0.475 | 0.667 |
| Agitation/aggression | 0.784 | −1.827 | 3.396 | 0.556 | 0.667 |
| Depression/dysphoria | −1.043 | −3.478 | 1.392 | 0.401 | 0.667 |
| Anxiety | 2.730 | 0.141 | 5.319 | 0.039 | 0.232 |
| Euphoria/elation | 0.974 | −3.396 | 5.344 | 0.662 | 0.722 |
| Apathy/indifference | 1.238 | −0.443 | 2.920 | 0.149 | 0.357 |
| Disinhibition | 2.604 | −2.233 | 7.440 | 0.291 | 0.583 |
| Irritability/lability | −0.807 | −3.357 | 1.743 | 0.535 | 0.667 |
| Aberrant motor behavior | 2.243 | −0.157 | 4.643 | 0.067 | 0.268 |
| Nighttime behaviors | 2.293 | 0.195 | 4.391 | 0.032 | 0.232 |
| Appetite/eating changes | −0.291 | −2.695 | 2.114 | 0.813 | 0.813 |

**Panel C. Within-domain frequency-by-severity interaction terms**

| **NPI domain** | **B** | **95% CI lower** | **95% CI upper** | **P value** | **FDR q** |
| --- | --- | --- | --- | --- | --- |
| Delusions | −0.303 | −2.203 | 1.597 | 0.754 | 0.823 |
| Hallucinations | 0.666 | −1.939 | 3.271 | 0.616 | 0.740 |
| Agitation/aggression | 1.034 | −1.867 | 3.935 | 0.485 | 0.740 |
| Depression/dysphoria | −2.171 | −4.125 | −0.218 | 0.029 | 0.176 |
| Anxiety | −0.673 | −2.637 | 1.291 | 0.502 | 0.740 |
| Euphoria/elation | −1.634 | −7.248 | 3.980 | 0.568 | 0.740 |
| Apathy/indifference | −2.431 | −4.098 | −0.765 | 0.004 | 0.051 |
| Disinhibition | −1.617 | −5.818 | 2.585 | 0.451 | 0.740 |
| Irritability/lability | 0.249 | −2.287 | 2.785 | 0.848 | 0.848 |
| Aberrant motor behavior | −1.304 | −3.640 | 1.033 | 0.274 | 0.740 |
| Nighttime behaviors | 0.709 | −1.306 | 2.723 | 0.491 | 0.740 |
| Appetite/eating changes | −1.127 | −4.093 | 1.838 | 0.456 | 0.740 |
